# Freezing More than Gait: A Case Report of Freezing of Urination (FOU) in Parkinson's Disease

**DOI:** 10.1155/2020/5190703

**Published:** 2020-01-21

**Authors:** Mengyuan Xu, Tao Chen, Caifei Yang, Xu Meng, Qingyun Peng, Xiaoguang Lei

**Affiliations:** ^1^Department of Neurology, Kunming Medical University, Kunming 650000, China; ^2^Department of Neurology, First Affiliated Hospital of Kunming Medical University, Kunming 650000, China

## Abstract

Freezing of gait (FOG) is a disabling phenomenon that is described by patients with Parkinson's disease (PD). Not only gait may be involved in the freezing phenomenon, but also some nonmotor symptoms, such as freezing of urination (FOU) in this case. The characters of urinary dysfunctions in this case resemble “off” freezing: (1) abrupt difficulty in starting or continuing in urination; (2) the urinary dysfunctions fluctuated with medication state; and (3) the urinary dysfunctions could be alleviated dramatically by an external cueing. Urinary dysfunctions in this patient (and maybe more PD patients) are associated not only with the classical “nonmotor symptoms” but also the freezing phenomenon. FOU could be a part of the spectrum of freezing symptoms. The subtypes of the freezing phenomenon will shed light on the PD pathophysiology and clinical treatment.

## 1. Introduction

Freezing of gait (FOG) is a disabling phenomenon that is described by patients with Parkinson's disease (PD) as the sensation that both feet are suddenly glued to the floor making the next step impossible. The similar sudden and transient difficulty in starting or continuing rhythmic and repetitive movement is the phenomenon of “freezing.” Not only gait may be involved in the freezing phenomenon but also speech, handwriting, or even more [1].

Urinary symptoms frequently occur in patients with PD [2, 3] when severity of disease exceeds Hoehn and Yahr stage 3 [4]. However, there is no report about the urinary dysfunctions in PD which could fluctuate with medication state and exhibit a phenomenon similar to “freezing.” In this case report, we described a Chinese female PD patient, who was found to have freezing of urination (FOU).

## 2. Case Description

A 55‐year‐old Chinese woman with no significant past medical or family history developed progressive resting tremor on her right upper limb for approximately ten years. Difficulties were gradually associated with bradykinesia, gait, and postural instability. Brief episodes of movement breakdown, particularly when initiating walking or turning, have appeared 7 months ago and even caused falls several times, and the symptoms disappeared or alleviated obviously in on state. In the last three months, she complained of urinary dysfunctions as “she tried to urinate smoothly but it's too hard for her” and had to wear-and-take-off her pants to facilitate urination. Interestingly, just like “off” FOG, after intaking dopaminergic medications and turning in the on-state, her urinary dysfunctions were alleviated instantly (Table 1). She was diagnosed according to the MDS clinical diagnostic criteria for Parkinson's disease [5] two years ago and prescribed dopaminergic replacement medications simultaneously.

On examination, she exhibited right-limb resting tremor, bradykinesia and rigidity, a slow shuffling gait, postural instability, and FOG in off state. Her Hoehn and Yahr stage was 3 in off state. FOG was presented frequently when she tried to start walking or turn during off state (4.5 hours after intaking 500 mg levodopa) and disappeared during on state (1 hour after intaking 500 mg levodopa).

**Figure 1:**
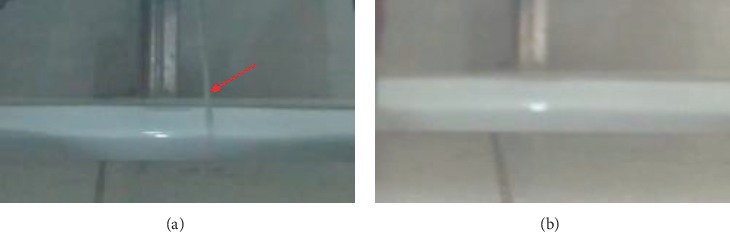
The process of urination recorded as a video. (a) The picture with a red arrow in the left represents that urination can be observed in the video recording and (b) the picture without an arrow in the right represents that urination cannot be observed through the video recording.

**Figure 2:**
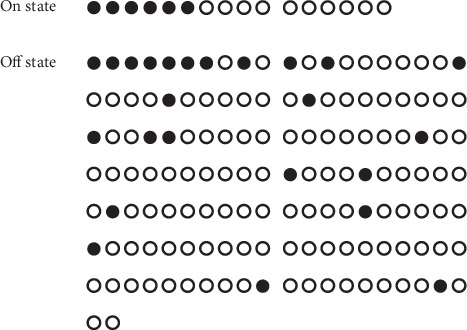
Different urination processes in on/off state. (a) one of the circles represents that the duration of urination lasted about 6 seconds (10 circles represent 1 minute, a row of circles in one line represents 2 minutes in total). (b) A black circle indicates that the urinary flow lasted less than 3 seconds (including no flow). (c) A white circle indicates that the urinary flow lasted more than 3 seconds.

Simultaneously, with the consent of the patient, one female physician accompanied the patient to void in a squatting pan in a private stall with a timer and camera, shooting exclusively on the urinary flow (without any body parts on her privacy). The subjective starting and ending time points in the urination process were recorded separately by the patient knocked on the section door, and the interval timing during on state (T-on) and off state (T-off) was counted by using a timer. The continuity of her urinary flow was extracted from the flow video (see Figure 1). The T-on period lasted 1 minute and 38 seconds, and the T-off period lasted 14 minutes and 14 seconds. The number of urination interruptions during T-on was 10 which is smaller than 118 during T-off (white circles in Figure 2).

## 3. Discussion

As one of the nonmotor symptoms, urinary dysfunction presents in approximately 30%–65% of PD patients [6]. Due to the detrusor overactivity caused by loss of dopaminergic neurons [7], most reported urinary symptoms are overactive bladder symptoms such as urinary urgency (77.3%) and nocturia (61.9%), which are described by patients that they often feel a sudden urge to urinate and wake several times during night for urination [6, 8].

In this case, the patient had different urinary symptoms (Table 1). The reasons we name it freezing of urination (FOU) are that they are sudden and transient difficulty in starting or continuing (urinary) movement, which are similar to the phenomenon of “freezing” [1].

Like FOG, which is traditionally classified into off-state freezing of gait (“off” FOG) phenotype and on-state freezing of gait (“on” FOG) phenotype [9, 10], FOU may also be observed in different phenotypes. For this patient, we thought she had “off” freezing of urination.

The “Off” freezing phenomenon is more commonly observed than the “on” freezing phenomenon [11, 12]. Despite pharmacotherapies have been shown to help with “freezing” of gait [1, 13], the effects of levodopa treatment in two phenotypes of FOG (“on” or “off” FOG) are different: levodopa could reduce the frequency and duration in “off” FOG but worsen “on” FOG conversely [9]. The FOU in this patient resembles “off” freezing because her symptoms had fluctuated with medication state and deteriorated during off state, and its frequency and duration can also be reduced by intaking levodopa as same as “off” FOG.

In addition, as FOG could be largely influenced by task-specific external cueing [14], the urinary dysfunctions in this patient can also be alleviated dramatically by a special cueing: when she felt the process of urination stopped but not incomplete, she had to wear-and-take-off her pants repeatedly to facilitate the following urination. This behaviour is very useful for her to end the urination process and to restart a new cycle. We also tested if the sound of running water or speech cueing could help her, but neither of them worked.

However, we admitted that freezing and bradykinesia are all involved in the urinary dysfunction in the patient, just like they are involved in gait impairments. In comparison with freezing, transient freezing phenomenon lasts only a few seconds and less than 1 minute [9], and bradykinesia slowers the speed of urination (weak stream) and is always present to some degree [1], that is why the “weak steam” in this case can be improved by supplement of external dopamine but cannot be eliminated completely [15, 16].

To conclude, the characters of urinary dysfunctions in this patient involved (1) abrupt difficulty in starting or continuing the urination which is one of the basic characters in “freezing”; (2) The urinary dysfunctions fluctuated with medication state, resembling “off” freezing; (3) The urinary dysfunctions could be alleviated dramatically by an external cueing. All of these remind us that urinary dysfunctions in this patient (and maybe more PD patients) are associated not only with the classical “nonmotor symptoms” but also with the freezing phenomenon. FOU could be a part of the spectrum of freezing symptoms.

Is there a phenomenon of “on” freezing of urinary dysfunctions we never observed? Are defecate or other motor or nonmotor symptoms involved in the freezing phenomenon? These questions are needed to be answered in the future. We propose that the subtypes of the freezing phenomenon will shed light on the PD pathophysiology and clinical treatment.

## Figures and Tables

**Table 1:** Urinary symptoms during off/on state.

Urinary symptoms	Off	On
Incomplete emptying	+++	+
Frequent urination within 2 hours	+++	−
Intermittency	+++	+
Weak stream	+++	+
Durations of urination >5 minutes	+++	−

− = no symptom, + = mild, ++ = moderate, +++ = severe.

## Data Availability

The data used to support the findings of this study are available from the corresponding author upon request.
